# Microbial Dark Matter: from Discovery to Applications

**DOI:** 10.1016/j.gpb.2022.02.007

**Published:** 2022-04-26

**Authors:** Yuguo Zha, Hui Chong, Pengshuo Yang, Kang Ning

**Affiliations:** MOE Key Laboratory of Molecular Biophysics, Hubei Key Laboratory of Bioinformatics and Molecular-imaging, Center of Artificial Intelligence Biology, Department of Bioinformatics and Systems Biology, College of Life Science and Technology, Huazhong University of Science and Technology, Wuhan 430074, China

**Keywords:** Microbiome, Dark matter, Artificial intelligence, Knowledge discovery, Application

## Abstract

With the rapid increase of the **microbiome** samples and sequencing data, more and more knowledge about microbial communities has been gained. However, there is still much more to learn about microbial communities, including billions of novel species and genes, as well as countless spatiotemporal dynamic patterns within the microbial communities, which together form the microbial **dark matter**. In this work, we summarized the dark matter in microbiome research and reviewed current data mining methods, especially **artificial intelligence** (AI) methods, for different types of **knowledge discovery** from microbial dark matter. We also provided case studies on using AI methods for microbiome data mining and knowledge discovery. In summary, we view microbial dark matter not as a problem to be solved but as an opportunity for AI methods to explore, with the goal of advancing our understanding of microbial communities, as well as developing better solutions to global concerns about human health and the environment.

## Introduction

**Figure 1:**
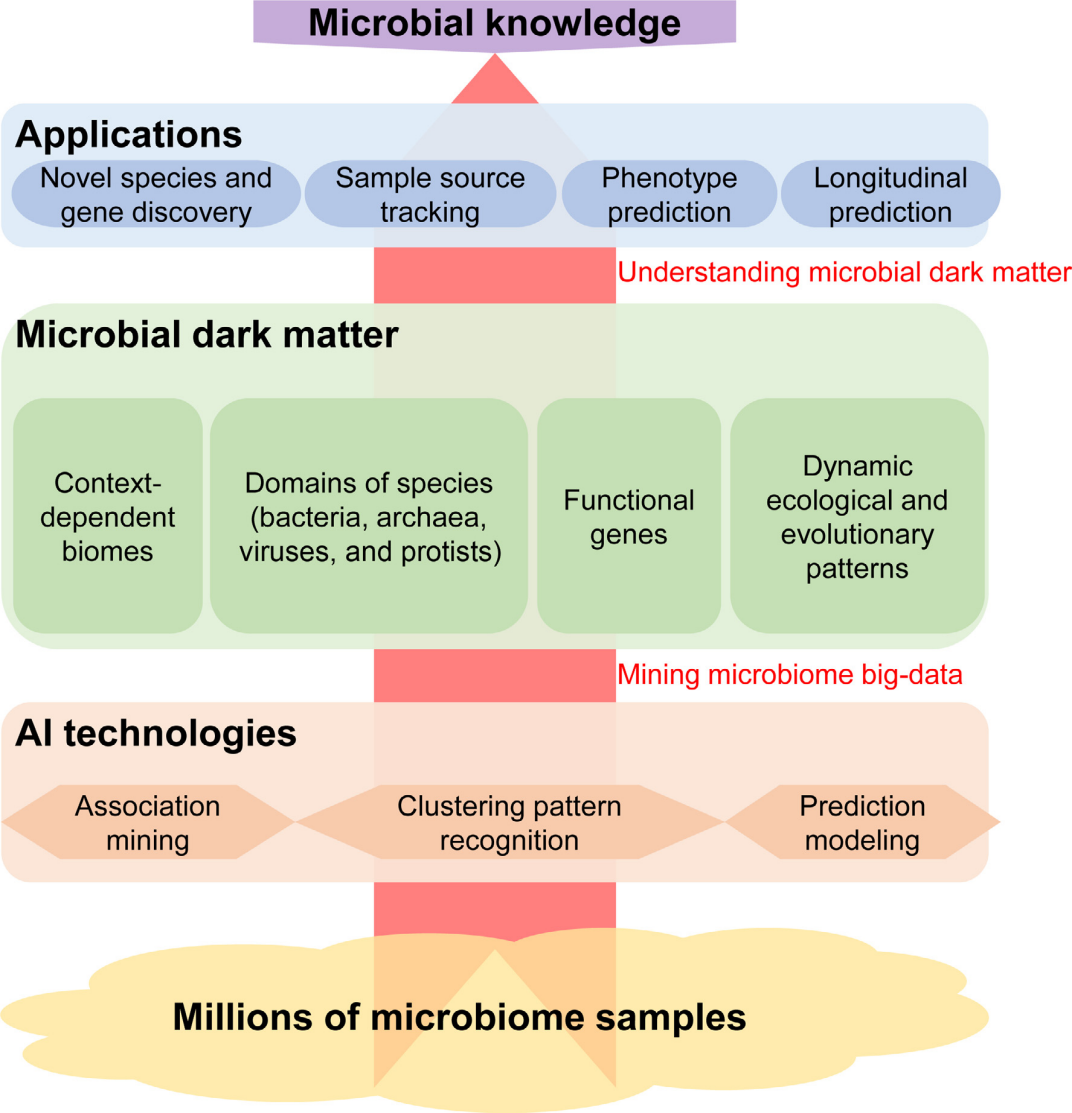
**The microbial dark matter and the techniques to better understand such dark matter toward better solutions in applications** There are three key steps for microbiome knowledge discovery from millions of microbiome samples, including the development of AI technologies and microbiome analysis tools, the sets of microbial dark matter to be unearthed, and countless applications. Among these, the microbial dark matter represents the core resource to be discovered. The major types of microbial dark matter introduced in this review include: more than a million context-dependent biomes in which microbial communities could reside; more than a million species, including bacteria, archaea, viruses, and protists; more than a billion functional genes; and the countless number of dynamic ecological and evolutionary patterns. AI, artificial intelligence.

Microbial communities from diverse global environments have been investigated, revealing abundant novel species and genes, in addition to unique spatiotemporal dynamics across environments [1], [2], [3]. Nevertheless, a substantial amount of microbial biodiversity remains to be discovered. These novel community structures and functions constitute an enormous reservoir of diversity that has been referred to as microbial dark matter. Microbial dark matter comprises several different components (Figure 1). 1) There are millions of biomes (niches) that microbial communities inhabit [4], [5], [6], including general environments such as freshwaters and soils, in addition to context-dependent biomes or understudied biomes such as the gut microbiomes of patients with different diseases. 2) In addition, tens of millions of microbial species are known that span several life kingdoms, including bacteria [1], [3], [7], archaea [2], [8], viruses [9], [10], [11], [12], [13], [14], and protists [15]. 3) Furthermore, billions of functional genes are encoded by genomes within microbial communities [2], [16], [17]. 4) Finally, there are countless dynamic ecological and evolutionary patterns that influence microbial community compositions [15], [18], [19], [20], [21], [22]. All of these areas of microbial dark matter hold great potential for a better understanding of the microbial world, but many of these areas remain understudied [7].

Big data and artificial intelligence (AI) technologies have enabled a more efficient mining of microbial dark matter to generate a better understanding of microbial communities and their potential applications [7], [23]. Microbiome big data are derived from millions of microbial community samples, wherein each sample could comprise a few hundred megabytes of 16S rRNA gene sequencing data. In addition, whole-genome sequencing (WGS) can yield over 10 gigabytes of sequencing data per sample. Thus, a typical study including a few thousand samples could comprise over 10 terabytes of sequencing data [24], [25]. AI refers to computer systems that leverage computers and machines to mimic the problem-solving and decision-making capabilities of the human mind. In this manuscript, AI includes deep learning, and AI methods in this context mainly refer to methods that use deep learning (*e.g*., neural networks). Typical AI technologies used in microbiome big data analyses include those for association mining, cluster pattern recognition, and prediction modeling [26], [27], [28], [29], [30], [31] (Figure 1). Mining of microbiome big data can help generate knowledge and models for many applications, including the discovery of novel species and genes, sample source tracking, phenotype prediction (especially for disease diagnosis), and prediction models for longitudinal studies.

In the following text, we first introduce big data and AI technologies used for microbial dark matter analysis. We then introduce the primary types of microbial dark matter that are investigated along with current computational solutions for mining such dark matter. Representative studies are also highlighted that have leveraged AI technologies to generate profound insights across a broad spectrum of applications. Finally, we summarize the advantages of AI technologies in solving microbial dark matter problems, while also describing current bottlenecks and possible future solutions for microbial dark matter mining.

## Microbiome big data + AI: venue for microbiome knowledge discovery

The rapidly increasing number of microbiome samples from a variety of global environments (also referred to as biomes), in addition to the massively increased level of sequencing data generated from these samples, has led to the formation of microbiome big data, which represents an important resource pool for knowledge discovery [7], [24]. Concomitantly, AI has become an important method, if not the most important method, for mining microbiome big data to generate deeper understanding of microbial communities [23], [27], [29], [30], [31], [32]. Indeed, data integration and data mining are two key modules in most microbiome analyses, although these modules are context-dependent in different analytical applications.

There are currently several databases available for microbiome data integration, including specialized databases such as MetaGenomic Rapid Annotations using Subsystems Technology (MG-RAST) [33], European Bioinformatics Institute (EBI) MGnify [4], and Qiita [34], in addition to general databases like the National Center for Biotechnology Information (NCBI) Sequence Read Archive (SRA) [35], [36], [37]. MG-RAST is a public resource for automated phylogenetic and functional analysis of metagenomes. It produces automated functional assignments for metagenomic sequences by comparison against both protein and nucleotide databases. Likewise, Qiita is an open-source web-based platform that enables non-bioinformaticians to easily conduct their own analyses and meta-analyses. Among these databases, the EBI MGnify database is a typical data resource for microbiome studies [4], containing sub-millions of microbiome samples and their sequencing data, as well as analytical results associated with samples. Nearly a million microbiome samples, sequencing data, and meta-data (*e.g*., data for environmental factors, phenotypic characteristics, and other features) have already been deposited in these databases, and most are publicly available, thereby representing an enormous pool of resources for knowledge discovery [4].

There are currently hundreds of tools for microbiome data analysis in microbiome data mining that can be used for different analytical approaches and at different stages. For example, Mothur [38] is a popular traditional tool for the quality control of 16S rRNA sequencing data, while QIIME 2 [39] is also a widely used traditional tool for microbial community structure profiling. Likewise, many more analytical tools are available for functional profiling of microbial communities. These tools enable the rapid transformation of microbiome sequencing data to community structures and functional profiles [24]. In addition, more advanced traditional analytical tools are also available, including HUMAnN2 [40] and MetaPhlAn2 [41] that enable in-depth analysis of community functions. Further, the traditional bayesian-based method SourceTracker [5] can be used for microbial source tracking, while the traditional method antiSMASH [42] and the AI method DeepARG [43] can be used to mine functional genes.

However, the currently available millions of microbiome samples across hundreds of global environments have led to a shortage of AI methods for mining this volume of data, either for sample comparison and source tracking, functional gene mining, or discovery of dynamic patterns. 1) Current methods in sample comparison and source tracking [5], [6] are either based on distance calculations or unsupervised learning and exhibit a tradeoff between accuracy and efficiency, wherein they are only able to accurately source track a few tens of samples. 2) Current methods in functional gene mining [23], [42], [44] are based on database searches that are not able to find novel genes, while reference-free methods have a high false-positive rate. 3) Current methods and pipelines for context-dependent analytical applications are also limited by their inability to mine intrinsic patterns hidden among thousands to millions of samples [18], [20], [22], [45], [46]. All of these limitations have necessitated the development of AI methods that could help in microbial dark matter knowledge discovery.

## Dark matter in the microbiome and the computational mining techniques

### Context-dependent biomes

Hundreds of biomes, and countless context-dependent biomes, have been annotated or investigated [4]. These include hundreds of general biomes (*e.g*., soils and freshwaters) and context-dependent biomes that are more specific than general biomes [4]. Context-dependent biomes are involved in many concrete applications related to microbiome knowledge, such as population-specific [1], [19] and disease-related patterns [47], [48].

Although context-dependent biomes are directly related to various microbiome applications, most remain understudied [1], [49]. For example, it remains unknown how gut microbial communities can reflect the progression of colorectal cancer (CRC) in patients [47], [48]. Current studies have only indicated that gut microbial communities change with CRC progression. Although there is evidence that gut microbiota could be used to diagnose CRC, it is still not mature for using gut microbial communities as indicators for CRC progression. This is partially due to a lack of understanding of how gut microbial communities mediate CRC, in addition to the lack of an accurate model for prediction. These problems are exacerbated due to the limited accuracy of many disease models because of regional variation [50] or are confounded by host variables such as body mass index (BMI) and age [51].

Numerous gut microbial community datasets have been accumulated, despite that gut microbiomes remain largely understudied, and these datasets are numerous and diverse enough to enable accurate predictions. Indeed, this high abundance of gut microbiome datasets has been useful for microbial source tracking [52], [53], [54], [55], [56]. The SourceTracker program uses a bayesian approach and has been used to differentiate samples from the human mouth, gut, and skin, in addition to monitoring the progression of gut microbial community development in infants [21]. The random forest approach is more widely used to identify microbial community sources via application toward the prediction of locations and times for forensic studies [7], [57], [58], in addition to application in predicting sources of contamination [59], [60]. ONN4MST [61] is a deep learning method which employs a neural network model to source track microbial communities at high efficiency and accuracy without any prior knowledge about the microbial communities to be estimated. Its pre-built biome ontology includes 60 environmental biomes, 25 host-associated biomes, and 10 engineered biomes, which represent the most comprehensive potential sources utilized for source tracking. However, ONN4MST is limited to searching biomes contained in the pre-built biome ontology, but cannot search understudied biomes. EXPERT [62] is also a deep learning method for microbial source tracking, which employs neural network models and acquired flexibility by applying a transfer learning approach, enabling adaptation to newly introduced biomes. It pre-built three neural network models for source tracking among 1) all possible sources from diverse environments, 2) human-associated sources, and 3) human gut-associated sources. Therefore, EXPERT enabled source tracking in many related contexts, such as characterizing disease or time-related compositional shifts of the human gut microbiome. These methods and tasks can contribute to a better understanding of microbial communities. Several databases and data mining methods have been previously reported for microbial source tracking, with representative databases and analytical methods shown in Table 1.

**Table 1:** **Microbiome sample source tracking methods**

**Method type**	**Algorithm**	**Data pre-processing**	**Computational model**	**Computational resource**	**Representative tool**	**Ref.**
Distance-based	Pair-wise sample distance or similarity	No feature selection	Pair-wise distance calculation	Multi-thread, GPU acceleration	JSD	[52]
					UniFrac	[53]
					Meta-Storms	[54]
					Meta-Prism	[55]
Unsupervised	Bayesian; EM	No feature selection	Model-free unsupervised learning	Multi-thread	SourceTracker	[5]
					FEAST	[6]
Supervised	Ensemble learning; deep learning	Feature selection before source tracking	Model-based supervised learning	Multi-thread, GPU acceleration	Random forest	[56]
					ONN4MST	[61]
					EXPERT	[62]

*Note*: GPU, graphics processing unit; EM, expectation maximization.

### Domains of species

Traditional microbiome studies have primarily focused on bacteria, although bacteria only represent a small fraction of all microorganisms. In addition to bacteria, archaea, viruses, and protists are also often abundant in environments. Archaea have distinct molecular characteristics from bacteria, despite often being considered “prokaryotes”. Archaea are commonly found in extreme environments and define the limits of life on Earth in many cases [63]. Archaea were originally discovered and described in extreme environments including in high salinity [64], extremely acidic [65], and anaerobic environments [66]. Many unique archaeal genes have been implicated in the adaptations to these extreme environments [65], [66]. In addition, viruses are not strictly defined as microbial organisms, because they only harbor a small number of genes and are surrounded by a protein coat. Viruses, as very small infectious agents, rely on living cells to multiply and are the smallest and most abundant of all microorganisms [67]. Protists are unicellular eukaryotic microorganisms that exhibit less complex physiological structures than other eukaryotes. Protists are not necessarily phylogenetically similar but are considered a single group because they do not fit into other taxonomic kingdoms [68].

All microorganisms, including bacteria, archaea, viruses, and protists, are representatives of billions of years of evolution, in addition to the adaptations required to live in specific environments [69]. For example, phylogenetic analyses of these microorganisms have revealed that the composition of human gut microbiomes is affected by hosts [70], while additional research has illustrated dynamic changes and the robustness of gut microbiota in the adaptations to their hosts [71]. Although microorganisms harbor very important functional genes, most of their genomic contents remain poorly understood. For example, several families of archaea were only recently characterized, with their novel evolutionary positions only being recently determined, while phylogenetic positions of most protists have yet to be determined [72]. Moreover, over 60,000 protistan species have been identified in the NCBI taxonomy system, while many have also yet to be identified [73]. Deeper insights into these poorly understood taxa could reveal an enormous amount of important, but currently unknown genes. Several databases and data mining methods have been previously reported for the analysis of bacteria, archaea, viruses, and protists [4], [33], [74], [75], [76], [77], [78], [79], with representative databases and analytical methods shown in Table 2.

**Table 2:** **Databases and methods for the analysis of bacteria, archaea, viruses, and protists**

**Domainof species**	**Type**	**Name**	**Description**	**Website**	**Ref.**
Bacteria and archaea	Database	EBI MGnify	A platform to submit, analyze, discover, and compare microbiome data	https://www.ebi.ac.uk/metagenomics/	[4]
	Database	MG-RAST	A metagenomics service for analysis of microbial community structure and function	https://www.mg-rast.org/	[126]
	Software	QIIME 2	A microbiome bioinformatics platform for processing and analyzing the microbiome	https://qiime2.org/	[74]
Virus	Database	Refseq	Virus genome annotation and curation	https://ftp.ncbi.nlm.nih.gov/genomes/	[75]
	Software	Virfinder	A *k*-mer-frequency-based tool for virus contig identification	https://github.com/jessieren/VirFinder	[76]
	Software	Virsorter	A tool designed to detect viral signals in these different types of microbial sequence data	https://github.com/simroux/VirSorter.git	[77]
Protist	Database	Protist Ribosomal Reference database	Provide a reference database of carefully annotated Protist genomes	https://pr2database.github.io/pr2database/index.html	[78]
	Software	OrthoDB	OrthoDB provides evolutionary and functional annotations of orthologs	https://www.orthodb.org	[79]

*Note*: EBI, European Bioinformatics Institute; MG-RAST, MetaGenomic Rapid Annotations using Subsystems Technology.

### Functional genes from microbial communities

Billions of functional genes have been annotated. In addition, advancements in sequencing technologies and the development of microbiome culture strategies have led to several microbiome projects that focus on distinct types of biomes. For example, the human microbiome project [1] for identifying the human gut microbiome, the Tara Oceans project [80] for identifying the global ocean microbiome, and the Earth microbiome project [2] that focuses on identifying global soil microbiomes. These projects have generated a massive number of microbial genomes and provide significant reservoirs of functional genes.

Some functional genes represent community-specific housekeeping genes. These genes are essential for individual microorganisms (*e.g*., genes responsible for DNA replication and RNA transcription that are present in almost all species) but are also necessary for the homeostasis of the entire microbial ecosystem. For example, genes that participate in the nitrogen-cycling process and carbon-cycling process in soil bacterial communities have been detected in all community members and identified as community-specific housekeeping genes [2]. Since nitrogen availability is one of the most common environmental limitations in soils ecosystem, these housekeeping genes could aid in the depletion of excess nitrogen and help degrade recalcitrant soil organic matter, thereby maintaining ecosystem homeostasis [81].

Many functional genes of microorganisms are niche-specific and play important roles in stabilizing microbial community structures, while providing insights into the adaptations of specific microbial populations [2], [81]. These genes may only exist in a specific biome but participate in important metabolic pathways, while allowing adaptations to environments by degrading harmful substances [82], adapting to external disturbances [22], and adaptation to hosts [83]. One example is metal resistance genes that are enriched in soil biomes. In soil biomes, metals are major abiotic stressors [84], and many soil taxa have developed full sets of functional genes to adapt to metal stress, such as energy metabolism, integral components of membranes, ion transport/chelation, protein/amino acid metabolism, carbohydrate/fatty acid metabolism, signal transduction, and DNA binding [85]. Another example is functional genes that facilitate cellular motility that are enriched in lake biomes. Cells in these environments live in highly fluid habitats, and functional genes that enable cellular motility (for example, flagellum-formation proteins) are enriched in community members of water biomes [86].

Current focuses on functional genes among microbial communities primarily include antibiotic resistance genes (ARGs) and biosynthetic gene clusters (BGCs). ARGs are critical for maintaining ecological stability within communities, especially by enabling the resistance to outside stresses. In addition, BGCs are directly associated with important metabolic products of communities. Existing tools for ARG mining include ARGMiner and DeepARG, which have provided both data resources and data analysis methods for ARG analysis [43], [87]. Many tools have been proposed to detect ARG sequences from genomic or metagenomic sequence libraries. For instance, ResFinder [88] and SEAR [89] both specifically predict plasmid-borne ARGs, while PATRIC [90] has been developed to identify ARGs that encode resistance to carbapenem, methicillin, and beta-lactam antibiotics. However, these tools exhibit limited efficiency or accuracy, especially for identifying novel ARGs. To better understand microbial functional genes and their effects on microbial communities and environments, more powerful tools using deep learning are urgently needed.

antiSMASH 6.0 is a commonly used tool for BGC data mining and is capable of providing microbial BGC resources for comparison, while also providing machine learning models for identifying novel BGCs from microbial communities [42]. Moreover, antiSMASH 6.0 features improved speed and interactive visualization functionalities that provide a more user-friendly BGC mining platform.

In addition, the functions of many genes identified in microbial communities are unknown. For example, a recent study of rumen metagenome-assembled genomes identified 3535 potentially new species and a total of 442,917 encoded proteins involved in carbohydrate metabolism [91]. Moreover, a recent study identified 13 novel TII-PKS BGCs that are uncommon but likely have high clinical medicinal value as bacterial BGCs [92]. Several databases and analytical methods have been proposed for the analysis of functional genes from microbial communities [93], [94], [95], [96], [97], [98], [99], [100], with representative databases and analytical methods shown in Table 3.

**Table 3:** **Databases and methods for the analysis of functional genes from microbial communities**

**Type**	**Name**	**Description**	**Website**	**Ref.**
Database	ARGminer	Antibiotic resistance gene database	https://bench.cs.vt.edu/argminer	[87]
	CARD	Comprehensive antibiotic resistance database	https://card.mcmaster.ca/	[93]
	SEED	A database to support effective comparative genome analysis	https://www.theseed.org/wiki/Home_of_the_SEED	[94]
	Pfam	A large collection of protein families	https://pfam.xfam.org/	[95]
	EggNOG	A database of orthology relationships, gene evolutionary histories, and functional annotations	https://eggnog5.embl.de/#/app/home	[96]
	Uniref	A database provides a comprehensive protein information	https://www.uniprot.org/help/uniref	[97]
	MetaCyc	A comprehensive reference database of metabolic pathways and enzymes from all domains of life	https://MetaCyc.org	[98]
	KEGG	Kyoto encyclopedia of genes and genomes	https://www.genodme.jp/kegg/	[99]
Software	DeepARG	A tool with a fully automated data analysis pipeline for antibiotic resistance annotation of raw metagenomic samples	https://bench.cs.vt.edu/deeparg	[43]
	AntiSMASH	A tool for the rapid genome-wide identification, annotation, and analysis of secondary metabolite biosynthesis gene clusters in bacterial and fungal genomes	https://antismash.secondarymetabolites.org/	[42]
	HUMAnN2	A pipeline for profiling the microbial pathways	https://huttenhower.sph.harvard.edu/humann2	[40]
	PICRUSt2	Provide information about the functional composition of sampled communities	https://github.com/picrust/picrust2	[100]

### Microbial ecological and evolutionary patterns

Niche-specific spatiotemporal dynamics within microbial communities, in addition to the consequences of these spatiotemporal dynamics on species evolution, are key determinants for the formation, development, stability, and dynamics of microbial communities [21], [101], [102], [103]. However, many microbial ecological and evolutionary patterns remain to be discovered.

For example, the discovery of human gut microbial community enterotypes has enabled hundreds of projects to determine the “stable” status of both human and animal gut microbial communities [71], [104], [105], [106], [107]. Further, the existence of enterotypes for all humans on Earth has only been recognized in the last 10 years, while such patterns dynamically change with environments and host diets [18], [20], [22], [46]. Variation in human gut microbial communities has also been extended to the analysis of animals, leading to the identification of variation in other types of gut microbial communities [105], [108].

**Figure 2:**
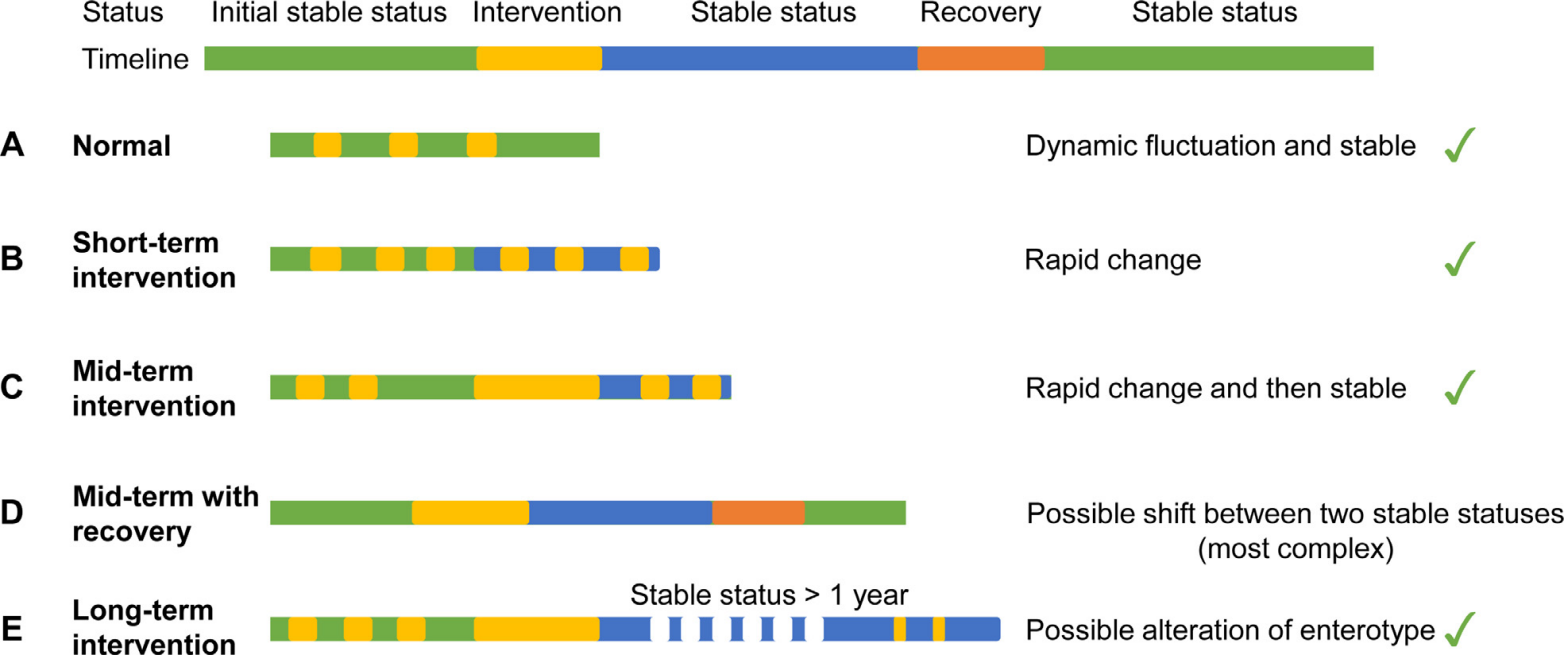
**The longitudinal dynamics of the human gut microbial communities have certain patterns** For short-term intervention, it has been demonstrated that dietary intervention is the main driver of the rapid change in the gut microbial community. For mid-term intervention, it has been demonstrated that the dietary intervention could become stable after a month. For long-term intervention, even the enterotype might be changed after one year. The dynamic patterns are based on human gut microbial community samples. And the community profile of each sample is based on the combination of species with different relative abundances.

Another example of ecological patterns in microbial community analyses is the temporal dynamics of human gut microbial communities. Human gut microbiota rapidly responds to changes in diet [103], [109], [110], and the composition of an individual’s gut microbiota is predominantly determined by dietary habits over the long term (*i.e*., more than 1 year) [107], [111]. However, these dynamics are highly variable among individuals [112], [113]. Over short-term time scales (*i.e*., less than 1 month), human gut microbiota can drastically change during dietary shifts, while such changes can also be quickly reversed after shifts in diets [22]. In addition, strong “plastic” patterns can be observed over mid-term time scales (*i.e*., between a month and a year) [22] (Figure 2).

However, these examples only represent a few of the many ecological and evolutionary patterns that remain to be discovered. For example, context-dependent patterns such as microbiome-related disease patterns [114] remain understudied, especially for cancer disease-microbiome patterns [115], longitudinal microbiome patterns [21], large-scale context-independent ecological patterns [2], and the evolutionary patterns of specific genes [116]. Among these areas, cancer disease-microbiome patterns are of particular importance [115], because they could provide evidence for the roles of bacteria in cancer states. Such studies could lead the way for the next generation of cancer prediction and therapeutic strategies [117]. Another area that lacks resolution is a basic and theoretical framework for how various genes act in concert to enable microorganisms to inhabit specific niches and how they may correspond to changes in the environment [116]. Homogeneous selection, homogeneous dispersal, and neutral theory are all ecological concepts that may help our understanding of these processes [118]. Likewise, the evolutionary process of genetic drift, natural selection, and homologous recombination may aid in developing the aforementioned framework [119]. Overall, investigations into these problems could help develop a better understanding of the ecological and evolutionary patterns ranging from small to large scales [22], [107], [111], [112], [113], [114], [115], [116], [117], [120].

## The dilemma of traditional methods could be solved by deep learning methods

Several computational solutions have been proposed to solve issues in understanding microbial dark matter [1], [2], [3] (Figure 1). However, most of these methods have tradeoffs and especially when considering big data analytical efficiency and accuracy. For example, traditional unsupervised learning methods such as SourceTracker [5] and FEAST [6] can achieve very high accuracy in microbial community source tracking when there are hundreds of samples and a handful of biomes. However, when the number of samples and biomes increases, running time increases rapidly, preventing large-scale source tracking. This problem could be solved by deep learning solutions by utilizing model-based methods such as neural networks that would enable improvements in both speed and accuracy during source tracking [61], [62].

Another example of a useful application of deep learning is in ARG mining, in which traditional methods based on Basic Local Alignment Search Tool (BLAST) searches have been used to identify candidate ARGs. However, such an approach is limited to comparison against known ARGs, and search speed is not very fast when using millions of candidates that require screening. The use of deep learning approaches via model-based methods has been shown to more efficiently mine novel ARGs out of millions of candidates [43], [121].

The abovementioned limitations suggest that AI techniques could be used to more efficiently uncover knowledge about microbial dark matter. AI techniques are advantageous in that they generate models from a large number of samples that are representative of global profiles within context-dependent subjects [27]. AI techniques are therefore suitable for accurate and fast searches when new sample (either a community, a gene, or a pattern) is searched against established models [28], [30], [122]. Thus, AI techniques are especially useful for mining microbial dark matter data, particularly when trying to improve tradeoffs between accuracy and efficiency.

**Figure 3:**
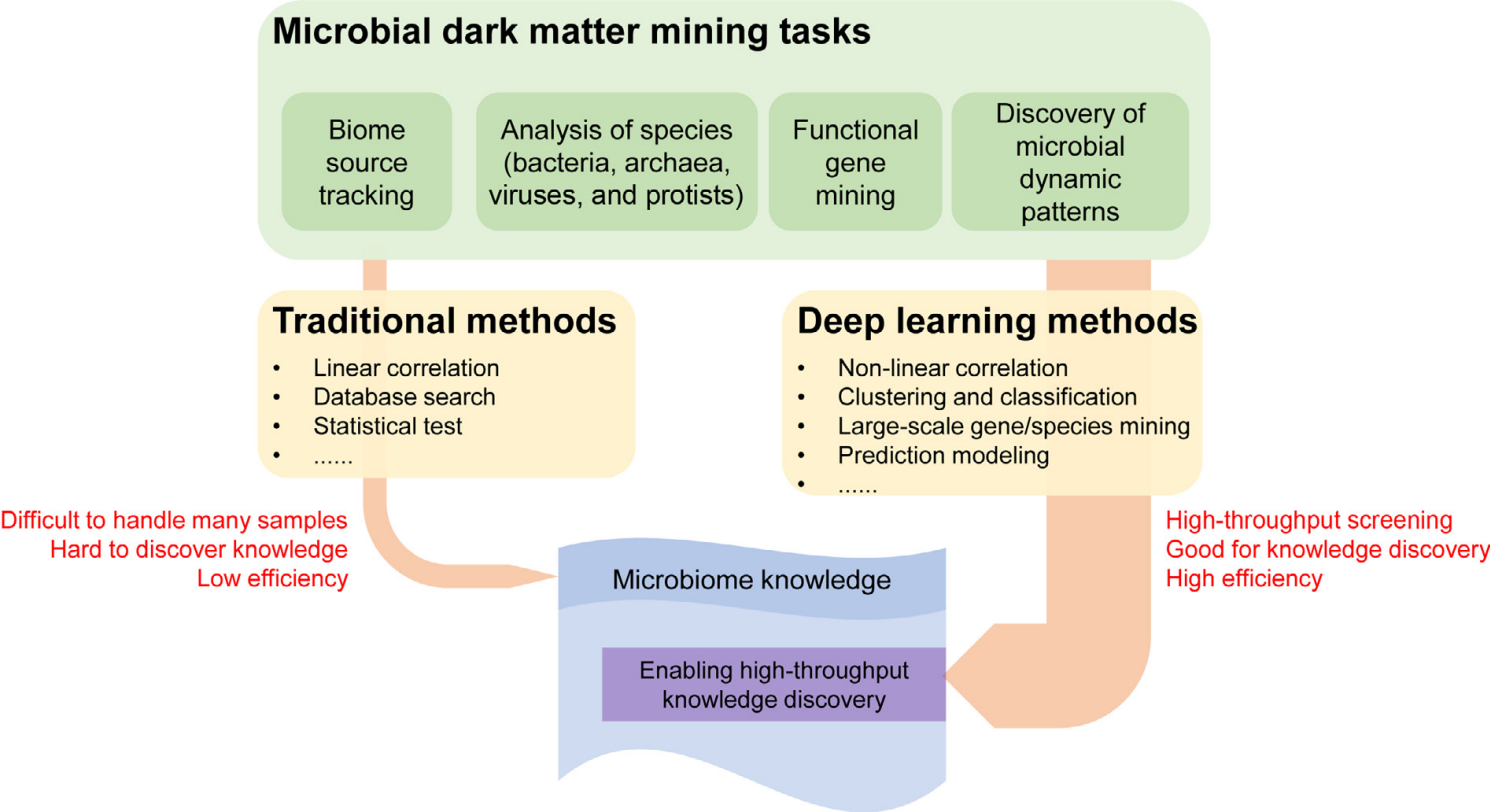
**The deep learning approaches for solving the microbial dark matter mining problems** Compared with traditional methods, deep learning methods have enabled high-throughput screening, thus is good for unknown knowledge discovery and has high efficiency.

Solutions for eliminating tradeoffs in current microbial data mining approaches rely on deep learning techniques [27], [28], [29], [30], [31] (Figure 3). In particular, model-based methods such as neural networks are advantageous in source tracking. For example, once a rational model has been built, improved efficiency and accuracy of model-based methods can be achieved that is comparable to, or even better than, existing distance-based and unsupervised methods [61], [123]. The same approach is suitable for gene mining issues [121]. In spatiotemporal dynamic pattern mining, deep learning approaches could also be used to discover intrinsic patterns out of cross-sections or longitudinal cohorts [124], [125].

One example application of the usefulness of these approaches is microbial source tracking. The first model-based method for source tracking, ONN4MST, already outperforms existing methods [61] for source tracking of known biomes. Further, the EXPERT method employs ONN4MST models to source track in different contexts [62] and has exhibited a high potential to facilitate mining of the microbial dark matter data. The EXPERT models are based on fundamental neural network models and transfer learning approaches, and exhibit high speed and accuracy, even when analyzing very few (a few hundred) samples from understudied biomes.

Functional gene mining from metagenome sequences is also an area that could be improved by AI approaches. For example, DeepARG uses a deep learning method that takes sequence alignment similarities as input and employs a neural network to enhance ARG prediction accuracy [43]. DeepARG can achieve a higher precision (0.97) and recall (0.91) than model-free ARG identification methods that exhibit a precision of 0.96 and a recall of 0.51. The hierarchical multi-task deep learning for annotating antibiotic resistance genes (HMD-ARG) method follows a similar approach by curating a comprehensive ARG database and then generating a hierarchical multi-task deep learning model that could help improve novel ARG discovery [121]. The supervised model-based methods are orders of magnitude faster than existing model-free methods.

Taken together, these studies have shown that supervised model-based methods are suitable for large-scale microbiome data mining and can facilitate accurate and efficient microbial dark matter discovery. Further, more advanced deep learning techniques such as convolutional neural networks (CNNs) and transfer learning could enable more accurate data mining, while also expanding the scope for knowledge discovery. Representative AI methods for the analysis of microbial dark matter are shown in Table 4.

**Table 4:** **Representative methods for the analysis of microbial dark matter**

**Microbial dark matter**	**Representative traditional method**	**Representative AI method**	**Summary**
Context-dependent biomes	SourceTracker [5], FEAST [6]	ONN4MST [61], EXPERT [62]	AI methods are especially suitable for source tracking among thousands to millions of samples in a fast and accurate manner
Domains of species	QIIME2 [74], Virfinder [76], OrthoDB [79]	\	Current methods for bacteria, archaea, virus, and protist analyses are limited to identifying known species
Functional genes	HUMAnN2 [40], antiSMASH [42]	DeepARG [43], HMD-ARG [121]	Current methods could identify novel genes, but with low speed and low fidelity
Dynamic ecological and evolutionary patterns	PCoA, MITRE [120]	\	Current methods are not sensitive to identifying the dynamic ecological and evolutionary patterns

*Note*: AI, artificial intelligence; “\”, not reported.

## Applications in microbial dark matter analysis

**Figure 4:**
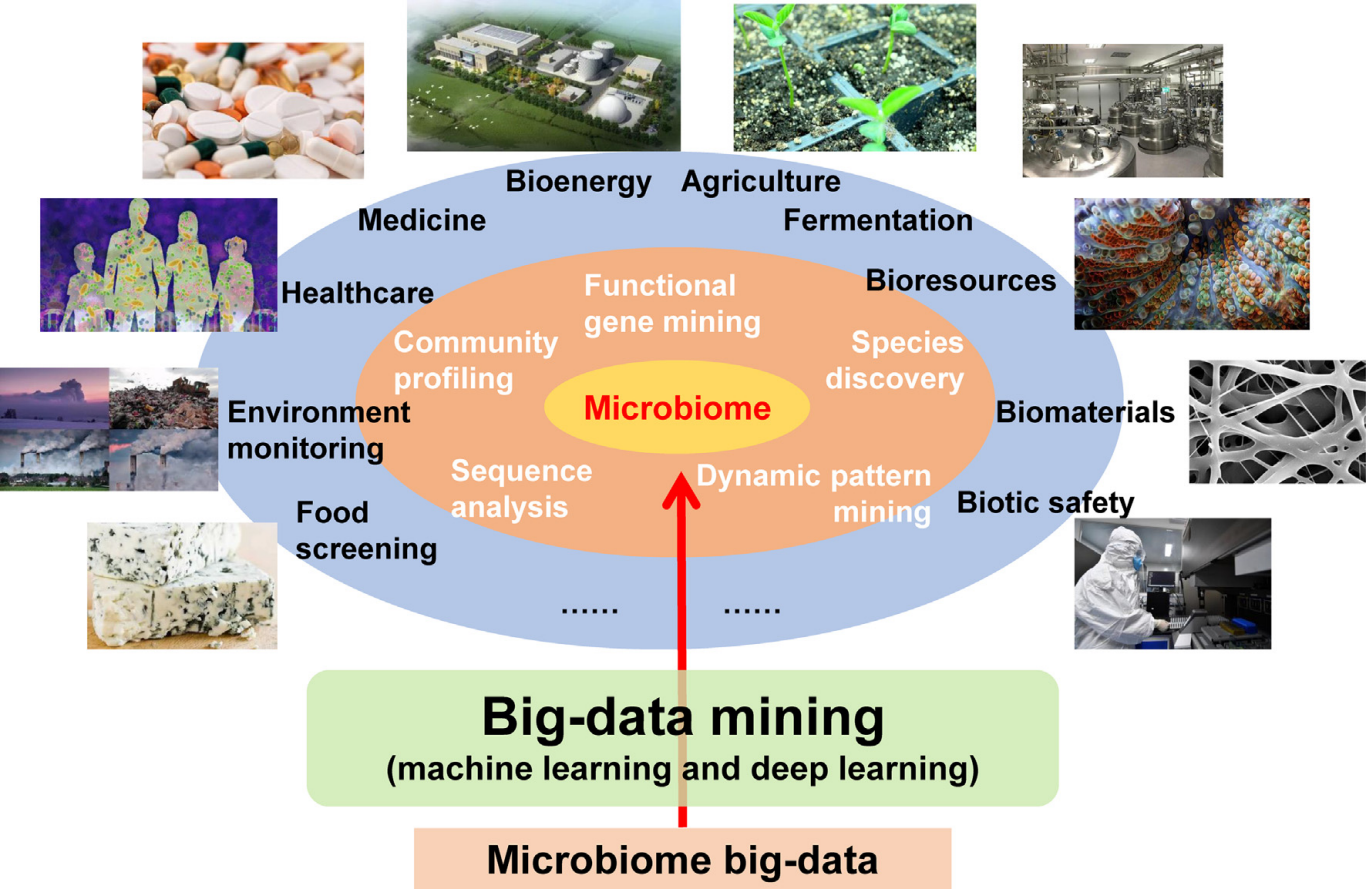
**Applications based on computational tools for microbial dark matter mining**

Computational tools, especially machine learning tools, have enabled a diverse set of applications that rely on microbial dark matter mining (Figure 4). These tools are described in detail below.

### Quality control of sequencing data

Genomic and metagenomic sequencing data commonly contain possible contamination from various environments, yet identification and removal of these contaminants remain difficult [126], [127], [128]. Machine learning-enabled source tracking and sequence clustering methods can act together to identify and remove contaminants, regardless of known or unknown sources [128]. Indeed, the application of machine learning methods to sequencing data can lead to the removal of most known contaminants [129]. Further, previously unknown or unexpected contaminants can also be identified and removed in an intelligent manner [130], enabling more accurate environmental and clinical data quality-filtering for subsequent studies [131]. For example, machine learning methods have enabled the accurate identification of contaminants in a typical molecular biology laboratory, including from workbenches and floors [6].

### Microbial source tracking

Microbial source tracking can be used in multi-faceted applications, including in contaminant sample source identification, forensic studies, and disease prediction [5], [6]. Traditional methods for microbial source tracking can generally be categorized into distance-based and unsupervised methods. Distance-based methods compute the distances between each pair of samples (using multiple distance measures) [52], [53], [54], [55], while unsupervised methods are limited by pre-defined sets of sources for source tracking [5], [6]. Supervised model-based methods [56] accurately quantify the contributions of source biomes for a specific sample but can also adapt to the analysis of samples from less studied biomes [61]. For example, the EXPERT method is able to accurately differentiate CRC stages in patients using a model built from more than 10,000 human microbiome samples from normal individuals [62].

### Novel species discovery from different domains of species

It has been estimated that there are more than a million unknown species and more than a billion unannotated microbial genes, providing an expansive opportunity for species and gene discovery. Novel species that live in extreme environments, or those that could generate important metabolites are of interest in clinical and industrial applications [132], [133], [134], [135]. Machine learning techniques have enabled novel species discovery from diverse taxa. For example, a recent study revealed thousands of novel protistan species at the global scale and established that protists are distributed discretely, with soil pH as the most important influencing factor on their distribution [73].

### Novel functional gene discovery

Functional gene mining from microbial communities, and especially ARG and BGC mining, are a focus of many studies [42], [136], [137]. Traditional methods for functional gene mining rely on databases comprising ARGs and BGCs that can be searched against, although these approaches are limited in the ability to discover novel functional genes [43], [87], [88], [89], [90], [136], [137]. Machine learning methods have, however, made it possible to discover novel functional genes more efficiently. For example, DeepARG has identified thousands of novel ARGs that were previously unannotated [43]. In addition, the use of HMD-ARG has shown that novel ARGs are functional via the combination of computation and wet-lab validation analyses [121].

### Phenotype prediction based on spatiotemporal patterns of the microbial communities

Human microbial communities are intricately linked with the health status of hosts, and it is possible to derive a model for predicting host phenotypes based on host-microbial communities [20]. Indeed, supervised learning methods are suitable for such analyses. For example, EXPERT has been used to accurately differentiate samples from patients with nearly twenty diseases, in addition to monitoring CRC stages in patients using models constructed from over 10,000 human microbiome samples in normal individuals [62].

Longitudinal predictions, as in the prediction of disease progressions, represent another potential area of application [21], [138]. Supervised learning has been successfully applied for longitudinal predictions by identifying key events along timelines, in addition to identifying differences among infants at different stages [62]. Machine learning methods have also been used for highly accurate human chronological age predictions [62]. Furthermore, machine learning methods such as random forest classification have been successfully applied in forensic studies leading to the ability to determine times and locations precisely [57]. Phenotype prediction modeling can also be used for identifying environmental indicators [139], [140]. For example, machine learning methods have been used to establish several microbial-based lake environment monitoring models using years of freshwater lake samples [139].

Overall, a more comprehensive understanding of microbial dark matter has opened the door for countless applications, with more in-depth applications being possible due to a better understanding of microbial communities. Although most of these applications are context-dependent, they could, in turn, provide large microbial community datasets that can help deepen our understanding of microbial communities across a variety of niches. Such iterative interactions between microbiome knowledge generation and applications could spur a significant improvement in microbiome research.

## Conclusion

Understanding microbial dark matter has emerged as a grand challenge for microbial research, and big data mining of microbial dark matter could be a powerful approach to understanding such dark matter. Microbial community niches, species, functional genes, and spatiotemporal dynamics all constitute important components of microbial dark matter. Microbiome studies have gradually produced an abundance of high-quality data that have enabled data mining techniques for large-scale microbiome data mining to promote an in-depth understanding of microbial communities. The rapid development of microbiome data mining could certainly boost the discovery of additional microbial resources and dynamic patterns from these dark matter datasets.

Current microbiome databases and analytical methods are suitable for small-scale microbiome data mining, but two important aspects of data analyses require urgent improvement. First, next-generation microbiome databases that contain sequencing data in addition to metadata, including environmental factors and phenotypic characteristics, are needed. The second is a need for enabling methods in genes mining among millions to billions of samples. Recent updates in metagenomic databases such as Qiita [34], in addition to model-based methods such as DeepARG [43] and EXPERT [6], have largely solved the large-scale microbiome data mining problem but are only appropriate for specific mining problems.

The potential insights from microbial dark matter discovery are indeed very inspiring. Microbial dark matter comprises novel biomes, species, functional genes, and spatiotemporal patterns. Increased discovery in these areas would certainly lead to the identification of new principles and could be useful for a tremendous number of applications in healthcare, biomedicine, environmental monitoring, and bio-safety, in addition to other areas.

Finally, we emphasize that it has already become clear that microbial communities have been collected from increasingly diverse niches around the world. Nevertheless, it is important to note that the currently sampled niches are far from complete, especially when considering the countless number of application-dependent contexts. Thus, microbial dark matter in a broad sense is almost infinite. However, data mining models could also be updated to cope with increasing diversity in dark matter, although multiple models might be needed to obtain optimal data mining results among different contexts. The arms race between microbial dark matter and AI modeling could lead to a much deeper understanding of microbial communities and their interactions with environments. In addition, it should be emphasized that advances in microbiome technologies will also play important roles in better understanding microbial dark matter. Recent advances in microbiome techniques include the use of third-generation sequencing (*e.g*., Oxford Nanopore) and the use of metatranscriptomics. For example, a recent study of *in vivo* dental plaques formed on hydroxyapatite disks for 6 h from 74 young adults documented the identification of 21 initial colonizing taxa based on full-length 16S rRNA gene sequences generated with long-read sequencing technology [141]. Metatranscriptomic sequencing can be used to ascertain a gene’s activity in a defined environment. Gosalbes et al. [142] conducted a metatranscriptomic analysis of fecal microbiomes from ten healthy humans and discovered that the gut microbiota’s primary functional roles were carbohydrate metabolism, energy production, and synthesis of cellular components. This work has proven that the metatranscriptome study could reveal the functions of microbes in amino acid and lipid metabolism.

Taken together, understanding microbial dark matter is not only a challenge, but also an opportunity for computational microbiologists to explore large datasets with the goal of better understanding microbial communities and identifying better solutions for current global concerns in human health and environments. AI technologies have already been applied to microbial dark matter mining problems, and we expect that the increased maturity of AI technologies will lead to increasing in-depth microbiome knowledge that could be mined out of the massive pool of microbial dark matter.

## CRediT author statement

**Yuguo Zha:** Writing - original draft, Writing - review & editing. **Hui Chong:** Writing - original draft. **Pengshuo Yang:** Writing - original draft. **Kang Ning:** Writing - review & editing, Conceptualization, Supervision, Funding acquisition. All authors have read and approved the final manuscript.

## Competing interests

The authors declare that they have no competing interests.
